# Cognitive and Affective Correlates of Temperament in Parkinson's Disease

**DOI:** 10.1155/2011/893873

**Published:** 2011-08-18

**Authors:** Graham Pluck, Richard G. Brown

**Affiliations:** ^1^Academic Clinical Psychiatry, The University of Sheffield, The Longley Centre, Norwood Grange Drive, Sheffield S5 7JT, UK; ^2^Institute of Psychiatry, King's College London, London SE5 8AF, UK

## Abstract

Parkinson's disease (PD) patients display low novelty seeking scores on the Tridimensional Personality Questionnaire (TPQ), which may reflect the low dopamine function that characterises the disease. People with PD also display raised harm avoidance scores. Due to these and other observations, a “parkinsonian personality” has been suggested. However, little is known about how these features relate to cognitive and affective disorders, which are also common in PD. We examined links between TPQ scores and performance on an attentional orienting task in a sample of 20 people with PD. In addition, associations between TPQ and depression and anxiety scores were explored. It was found that novelty seeking scores were significantly correlated with a reaction time measure of attentional orienting to visual novelty. Harm avoidance scores were significantly correlated with anxiety, but not depression scores. These findings extend our understanding of how temperament interacts with cognitive and affective features of the disorder.

## 1. Introduction

Parkinson's disease (PD) is primarily considered a neurological disease that produces movement disorders. However, patients with PD also tend to show a range of cognitive and psychiatric symptoms. In addition, a particular “parkinsonian personality” has often been described, which appears to be premorbid to neurological symptoms [1, 2] and may therefore be a temperament feature of the disease. At present, the extent to which such personality features contribute to the cognitive and affective components of the disease is poorly understood.

The primary pathology in the brains of PD patients is the loss of dopamine-producing cells in the substantia nigra [3]. Physiologically, the importance of the substantia nigra is in its dopaminergic projections to the striatum, which is one of the three main dopamine systems in the brain [4]. In PD patients, dopamine levels in this area have been observed to be only 10% of the normal level [5]. Therefore, PD is often considered as a disease that provides a model of low dopamine function in the human brain. Consequently, PD is of particular interest when considering theories that include the functional significance of dopamine.

One such theory, Cloninger et al.'s psychobiological approach to personality [6–9], posits that dopamine systems in the brain are the biological substrate of the temperament trait of novelty seeking. This “tridimensional” approach to personality measurement also proposes two further traits; harm avoidance (linked to serotonin) and reward dependence (linked to noradrenalin) [6]. However, later factor analytic studies revealed a fourth minor factor called persistence, which had formally been part of reward dependence [8]. Following the confirmation of the genetic structure of these four temperament dimensions, additional personality features were identified by Cloninger and colleagues which mature in adulthood. In fact, three additional character dimensions have been proposed which are influenced by insight learning, these are self-directedness, cooperativeness, and self-transcendence [9]. This psychobiological model of temperament and character has continued to evolve and is supported from a range of clinical and neuroscientific studies [7]. In particular, physiology-based research has focused on the temperament dimensions of novelty seeking, harm avoidance, and reward dependence, due to their supposed genetic basis and neurochemical substrates. It is of course reductionist and an over simplification to equate a personality trait directly with a single neurotransmitter substance. There is a large degree of cross-over between the different circuits and systems in the brain, and highly complex neurotransmitter interactions at the cellular level [10]. Nevertheless, it is accepted that PD is primarily a dopamine deficiency disorder [11] and that novelty seeking is more closely linked to dopamine function than any of the other neurochemical systems [12]. Therefore, in regard to PD, it is the temperament trait of novelty seeking which has received particular research attention. Indeed, patients with PD have been shown to display significantly lower novelty seeking scores than disability matched patients [13]. In a follow-up study, the role of dopamine was confirmed by the finding that 18 F Dopa striatal uptake in PET scans correlated with novelty seeking scores in PD patients [14]. Further research has confirmed this link between low novelty seeking and PD [15, 16]. 

The distinctive personality of PD patients has been recognized for many years. As early as 1880, Charcot had described low motivation in patients with PD [17]. In addition, patients with PD have been described as displaying “premature social ageing.” This was based on the observation that many PD patients have few friends, reduced social involvement, few hobbies, and often prefer to spend time on solitary tasks [18, 19]. Furthermore, it has long been noted that PD patients are more likely to be nonsmokers than the general population. From this, it has been suggested that premorbidly, PD patients are less hedonistic or more self-controlled than the average person [2].

Cognitive impairments, particularly involving frontal lobe function are also widely described in PD [20, 21]. However, it is possible that the cognitive impairments are in part manifestations of the parkinsonian personality profile. Recently it has been noted that frontal lobe-associated cognitive task performance correlates with personality variables in patients with PD, and it has been suggested that both may reflect a common mechanism [22]. In this respect, novelty seeking may be of particular interest, as it is partly defined as “a heritable bias in the activation or initiation of behaviours such as frequent exploratory activity in response to novelty” [9]. It could therefore be suggested that novelty seeking would be associated with orientation and attention in cognitive tasks. Indeed, it has been shown that there are significant correlations between novelty seeking scores and performance of visual attention tasks in healthy individuals [23]. In particular, orientation to novelty has traditionally been considered as crucial to adaptation and action within a changing environment [24]. We may therefore hypothesise that impaired performance of cognitive tasks involving attention to novelty, will be linked to personality factors in patients with PD, in particular, novelty seeking. 

Affective disorders are also common in patients with PD. Anxiety has been shown to be more prominent in PD than in healthy samples [25]. However, it is unclear whether this is a response to, or a symptom of, the disease itself. While anxiety varies with motor fluctuations and correlates with disease progression [26], this could be viewed as either indicating a neurobiological or a reactive mechanism. In support of a neurobiological cause is the observation that high levels of anxiety are linked to a serotonin transporter gene polymorphism in patients with PD [27]. Higher levels of depression were also found to be linked to the gene polymorphism, highlighting the fact that anxiety in PD tends to be comorbid with depressive symptoms, and that both may have a serotonergic basis. 

Considering depression in PD, different studies have produced different estimates of its prevalence and a variety of theories are available to explain its occurrence. Prevalence estimates have varied between 4% and 70% [28]. It is known that serotonergic function is impaired in PD [29], and this is a likely neurochemical substrate of affective aspects of the disease [30]. For example, it has been shown using transcranial sonography that there are morphological changes to the serotonergic dorsal raphe in depressed but not nondepressed PD patients [31].

Serotonergic function is also thought to underlie Cloninger's temperament feature of harm avoidance. In addition to the previously described association between PD and low novelty seeking, it is perhaps not surprising then that a relationship between PD and high harm avoidance scores has also been described, an observation that the authors attribute to the presence of depression [32]. Indeed, harm avoidance scores have been found to positively correlate with depression severity in patients with PD [33]. Nevertheless, the relationship between harm avoidance and depression in PD is not well understood. 

In this investigation we sought to examine the relationship between the personality dimension of harm avoidance and affective symptoms in patients with PD. We hypothesised that within a sample of PD patients, harm avoidance scores would be correlated with depression and anxiety scores. Furthermore, to examine the relationship between visual attention and the trait of novelty seeking in the same sample, we developed a method to measure how novel visual events influence attention. This was an adaptation of the attentional cueing paradigm [34], a reaction time task often used in experimental psychology. In the standard version, arrowheads presented at the centre of a display facilitate response times to stimuli that later appear at the cued location. Although such tests are widely used, they do not involve orientation to visual novelty. We used a version that we adapted ourselves in which visual novelty was manipulated so that its influence on attention could be measured with reaction times. We hypothesised that within our sample of PD patients, the trait of novelty seeking would be correlated with task performance.

## 2. Method

### 2.1. Participants

A total of 20 PD patients participated in this study, 11 of these were female. All were patients of the National Hospital for Neurology and Neurosurgery in London, and the diagnoses of idiopathic PD were made by a consultant neurologist specialising in movement disorders. The mean age of the patients was 68.5 years (SD = 9.4). The sample had a mean Hoehn and Yahr [35] stage of 1.9 (range 1–4), indicating a wide range of disease progression. Thirteen healthy control subjects also participated; all were volunteers who responded to advertisements. Ten of the control subjects were female. The mean age of the control sample was 69.7 years (SD = 9.1). There was no significant difference between the patients and controls for age (*t*(31) = .38, *P* = .710).

### 2.2. Materials and Apparatus

For the assessment of personality, the Tridimensional Personality Questionnaire was employed [6]. This measures three personality dimensions, novelty seeking, harm avoidance, and reward dependence. The hospital anxiety and depression scale was employed to measure severity of affective symptoms [36]. Although this is a brief measure, it is well suited to the current study as it was originally developed for use with medical outpatient samples [37] and has been validated for use with PD patients [38]. To administer the experimental task, a laptop computer with a colour 12.1′′ LCD monitor was used. The experimental task was implemented with the Visual Basic programming language. Details of the task are given below.

### 2.3. The Experimental Task

**Figure 1:**
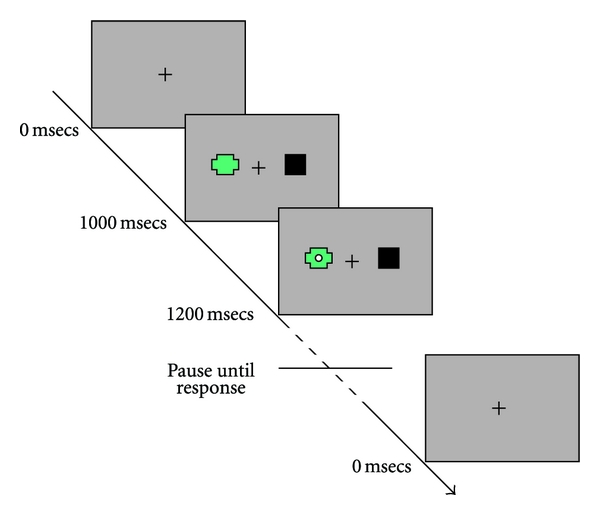
Representation of the sequence of events of a single trial in the novelty attention task.

To measure attention to novelty, participants were required to make a simple button press response to a white dot appearing on the laptop screen. This white dot appeared either to the left or right of a central cross. A pair of coloured shapes always appeared 200 msecs before the white dot, one on either side of the central cross (see Figure 1). These two coloured shapes appeared simultaneously and always in the same two locations. One of the shapes was always a light brown square. The other shape was varied such that a totally novel, different coloured shape, would often be substituted for the previous shape. The substitutions occurred randomly every four to seven trials. The coloured shapes always appeared as background to the target white dot. Therefore a typical trial involved the simultaneous display of two coloured shapes, one to the left and one to the right, followed one fifth of a second later by the target white dot, in front of one of the coloured shapes. The participants' task was to press the button as soon as they saw the white dot.

The location of the shape stimuli alternated randomly left to right, independently of the side that the target white dot would appear on. The shapes were therefore irrelevant to task performance, as shifting attention to either would confer no advantage in predicting the location of the white dot. However, if orientation of attention is influenced spontaneously then participants may orient their attention to the novel stimuli that is being displayed. If they did, and the white dot appeared there (200 msecs later), this might produce faster response times as their attention is already at the correct location. Conversely, if they spontaneously orient their attention to the novel stimuli (the coloured shape) and the white dot target appears on the other side (it is a 50 : 50 chance) then response times would likely be slowed as their attention has been diverted to the wrong location.

On 14% of the trials the target stimulus was not shown and a three-second delay was inserted before the next trial began. This was done to stop participants getting into the habit of just pressing the button each trial, as some were “blanks” they had to wait until the white dot appeared before pressing the button. If the response button was pressed on those “blank” trials where no target white dot was present, the computer emitted a tone and the word “error” was displayed on the screen.

After each trial the shape stimuli and white dot target disappeared and there was a one-second delay before the start of the next trial. Reaction times and number of errors were automatically recorded to a computer file. A visual representation of the temporal sequence of events in a typical trial is shown in Figure 1.

### 2.4. Procedure

All participants were contacted by telephone and an appointment made for their research participation. The assessment was conducted in the participant's own home. All participants contributed data on the novelty attention task performance; however, clinical data on affective symptoms and personality data was only collected on the PD patients. All participants provided informed and written consent, and the project was approved by the local research ethics committee.

PD patients were interviewed after an overnight withdrawal (approximately 11 hours) of their antiparkinsonian medication to ensure that they were in a hypodopaminergic state. During the interview, basic demographic and clinical information was collected. Next, the experimental task was administered. Participants sat approximately 70 cm from the laptop screen. In each location, dim lighting was employed to enhance the visibility of the display. One finger of the dominant hand was held over a microswitch and the participant was told to press the button as quickly as possible whenever they saw a white dot appear either to the left or right of the central cross. They were told to try and keep their fixation on this cross but that coloured shapes would appear in the background and that these would change occasionally. Responses were made via a button pad linked to a digital timing card in the computer. Input from the key was sampled at the rate of 1000 Hz. Two blocks of trials were performed. Each block involved 40 novel stimuli. There were 200 trials in each block. Each block took approximately 8 minutes to complete. Other cognitive assessments were performed which are not reported here.

### 2.5. Statistical Analyses

For all continuous data, the normality of distribution assumptions was verified with Kolmogorov-Smirnov one sample tests. Parametric tests were used with normally distributed variables and nonparametric equivalents when data was nonnormally distributed. To compare RTs on the novelty attention task, a mixed model ANOVA was used. For all statistically significant effects in the ANOVA calculations, estimates of effect size are provided as partial Eta^2^ statistics. Where *t*-tests were employed, effect sizes are reported as Cohen's *d*. To assess associations between variables and test our main hypotheses, Pearson bivariate correlation statistics were employed. For all inferential statistics, a value of *P* < .05 (two-tailed) was taken to indicate significance. All calculations were performed with PASW Statistics 18 [39].

## 3. Results

The novelty attention task is analysed first, followed by tridimensional personality questionnaire scores and hospital anxiety and depression scale scores. Finally, associations between the various measures are considered, as well as associations with disease progression.

For the novelty attention task, data from the first seven trials in each block were excluded, as they did not involve a novel change of stimulus. In addition, trials that occurred immediately after a withheld response were excluded. To control for anticipatory responding or lapses of attention, RTs of less than 100 msecs or more than 1000 msecs were excluded. From the remaining datasets, averages were calculated for the location of the target (either in conjunction with the repetitive or novel stimuli) and for the level of novelty. Level of novelty was defined as the number of times that the participant had seen the shape, including the current trial. Therefore, level of novelty ranged from 1–7, with 1 being a shape that was displayed for the first time. For each participant there were more data points for level of novelty 1–4 than for 5, 6, or 7. For this reason, mean rather than median averages were used to summarise the raw data as these are considered more appropriate for unequal datasets [40].

**Figure 2:**
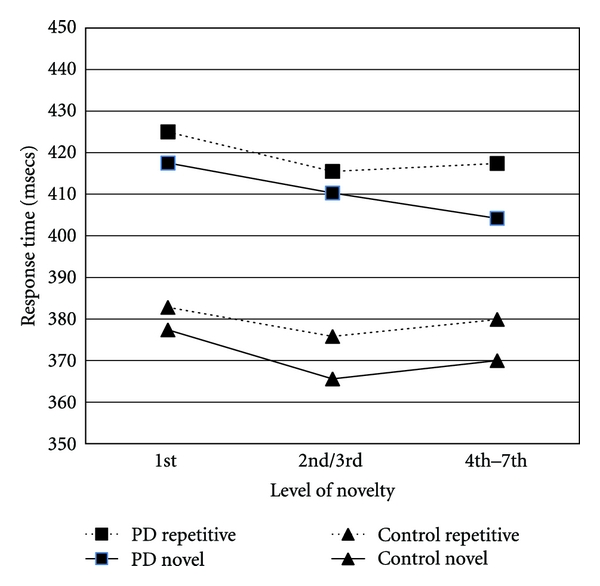
Comparison of the PD and control participants for response times when the target stimuli appeared in the repetitive or novel location and by how many times the novel stimuli had been presented (level of novelty).

Response times in all conditions for all participants are shown in Table 1. To analyse the effect of novelty on response times, data was entered into a mixed model ANOVA, with group as a between subjects factor, within subject factors were location (novel or repetitive) and level of novelty (how often the novel shape had been presented). In order to compare the effect of novelty, the data points were averaged to provide three main groups of novelty level, when the novel shape first appeared, the mean of the responses for the 2nd and 3rd presentation, and the mean of the 4th to 7th presentations. The response times using these groupings are shown in Figure 2 and were analysed as described above. There was no main effect of group (*F*(1,31) = 2.14, *P* = .154) and the interactions involving group membership were all non-significant. There was a main effect of location (*F*(1, 33) = 9.40, *P* = .004, partial Eta^2^ = .223) and of novelty (*F*(2,62) = 4.31, *P* = .018, partial Eta^2^ = .122). The first presentation resulted in response times approximately 9 msecs slower than either the second set (novelty level 2 and 3) or the third set (novelty level 4, 5, 6, and 7). The exact mean RT values in msecs were 408, 399, and 399, respectively. A planned contrast was performed to compare RTs when the novel stimulus was first presented with the combined 2nd and 3rd presentation RTs, the difference was statistically significant, *F*(1,31) = 9.26, *P* = .005, partial Eta^2^ = .230). However, the difference in RTs from the 1st presentation to the combined 4th, 5th, 6th, and 7th presentations was not statistically significant.

Absolute levels of errors (responding in the absence of a stimulus) were low with several participants making no errors at all, and so error distributions were nonnormal. For this reason, medians, rather than means, are reported. The PD sample made a median of 3 errors (range = 0–23) and the control group made a median of 4 errors (range = 0–9), this difference was not significant (Mann-Whitney *U* test, *P* = .896).

### 3.1. Anxiety-Depression Scores and Temperament

The PD sample had a mean novelty seeking score of 13.9 (SD = 4.8), the mean harm avoidance score was 18.7 (SD = 7.3), and the mean reward dependence score was 16.8 (SD = 4.0). On examination of the HADS scale scores, it was found that the mean score for depression was 4.8 (SD = 3.5, range = 1–15) and the mean score for anxiety was 7.3 (SD = 4.4, range = 2–19). Using the standard cut score of 8 [36], 3/20 (15%) of the PD patients would be considered as probable cases of depressive disorder. Similarly with the same cut score for anxiety, 8/20 (40%) of the PD patients would be considered as probable cases of anxiety disorder. Independent group *t*-tests revealed that there were no significant differences for anxiety or depression scores when male PD participants were compared with the female participants. Although there were no statistically significant sex differences for novelty seeking or reward dependence, it was found that the female PD patients had significantly higher levels of harm avoidance (female mean = 22.1, SD = 7.0, male mean = 14.4, SD = 5.5; *t*(18) = −.266, *P* = .016, *d* = 1.23). 

**Figure 3:**
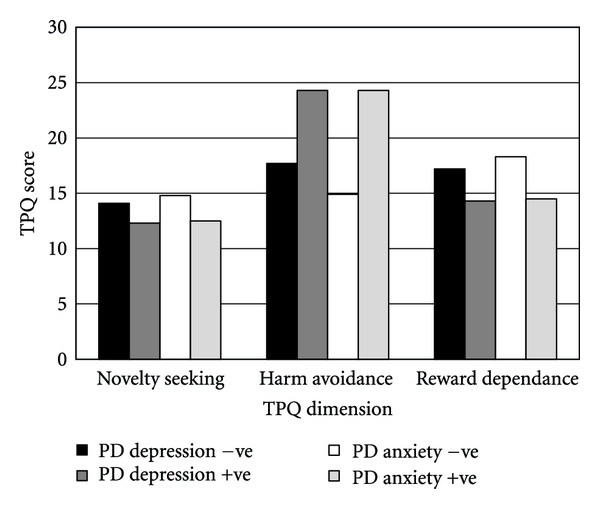
Comparison of Tridimensional Personality Questionnaire scores for PD participants with or without probable depression or anxiety.

To test our hypotheses that harm avoidance scores would be associated with depression and anxiety scores in the PD patients, correlation coefficients were calculated. These are shown in Table 2. It can be seen that there was a significant positive association between harm avoidance and anxiety severity scores; however, no significant association was detected with depression severity scores. There were no significant correlations between novelty seeking scores and depression or anxiety scores. The association between reward dependence and anxiety was approaching, but did not reach statistical significance. To further examine the relationship, those cases scoring above the cut scores for depression and anxiety were identified. Their TPQ scores are compared with those scoring below the cut scores in Figure 3. It can be seen that those PD participants scoring positive for probable anxiety disorder scored higher than the other patients for harm avoidance, and that this difference was statistically significant, *t*(18) = 3.53, *P* = .002, and *d* = .70. Furthermore, the same participants scored lower on reward dependence, a difference that was also statistically significant, *t*(18) = −2.37, *P* = .029, and *d* = 1.06. There was no significant difference for novelty seeking scores. A similar pattern of differences is seen when comparing those with and without probable depression. The three patients with probable depression appeared on visual inspection to score higher on harm avoidance and lower on reward dependence. However, no inferential statistical analysis was attempted due to the small sample size (3 verses 17). 

Returning to the novelty attention task, Figure 2 shows that there is a tendency for RTs to be larger for the 1st presentation of the novel stimuli than the combined 2nd and 3rd presentation RTs. This is true for the conditions in which the target stimuli appeared in either the repetitive or novel location, but particularly in the latter. Indeed, for the full sample, mean response times when the novel stimuli was first presented (in conjunction with the target) were approximately 9 msecs longer than for when it was presented the 2nd/3rd time (401.7 msecs (SD = 77) compared to 392.6 msecs (SD = 80)). As described above, this difference is statistically significant, and implies that the presence of a novel stimulus produces a measurable effect on responding. The difference between these two RTs could therefore indicate an overall effect of novel stimuli on responses. To test our hypothesis that attention to novelty would be correlated with novelty seeking, the difference between 1st presentation and the combined 2nd and 3rd presentation response times when the target appeared in conjunction with the novel stimuli were calculated for each patient. The correlations between this difference statistic and temperament dimensions on the TPQ are also shown in Table 2. It can be seen that there was a significant negative correlation between novelty seeking and the RT difference statistic, indicating that patients with high novelty seeking scores had smaller difference statistics. There were no significant correlations with the other temperament dimensions of harm avoidance and reward dependence.

Finally, the effect of disease severity on temperament, affective and cognitive performance scores was investigated. The median Hoehn and Yahr disease severity score [35] was 2 (range 1–4), this was used to divide the PD sample into those with relatively early progression (stages 1 and 1.5, unilateral symptoms only, *n* = 8) and those with more advanced disease (stages 2–4, bilateral symptoms, *n* = 12). It was found that there were no significant differences between the groups for any of the temperament dimensions, anxiety, depression, or the difference statistic used to measure the impact of novelty on attention. This was true even if a higher disease progression cutoff was selected which compared the six most advanced cases with the 14 less advanced cases.

## 4. Discussion

We report statistically significant cognitive and affective correlates of temperament dimensions in people with PD. Using the tridimensional personality questionnaire [6] we found that novelty seeking was significantly correlated with a measure of the effect of visual novelty on attention. In addition, harm avoidance was significantly correlated with anxiety scores. These two relationships were as hypothesised. However, we also hypothesised that depression would be correlated with harm avoidance, but this was not found to be so. The association between harm avoidance and anxiety was also confirmed with group mean comparisons. The PD participants with probable anxiety disorder had significantly higher harm avoidance scores than those without probable anxiety disorder. Using the same group comparison it was found that reward dependence scores were significantly lower in those PD participants with probable anxiety. None of the features studied in the participants with PD were found to be related to disease progression.

We found a relationship between the underlying concept embodied in the definition of novelty seeking and an experimentally derived measure of responses to visual novelty. We have previously shown that novelty seeking scores are associated with efficiency of parallel visual processing in a healthy control sample [23]. However, in the current study a cognitive association has been demonstrated which directly links to novelty, and in a sample of PD patients, individuals considered to be low on the trait of novelty seeking. Although in our own PD sample we found no specific evidence for low novelty seeking scores. Nevertheless, the finding of low novelty seeking among patients with PD has been demonstrated previously [13–16].

Using the custom-designed attentional task, it was found that both the PD and control participants displayed a significant novelty-related location effect. That is, responses were generally faster when the target appeared in conjunction with the novel stimulus. The procedure is therefore capable of measuring the influence of novel visual events on response times. It was also found that there was a significant level of novelty effect. That is, responses tended to be relatively faster on 2nd and 3rd (combined) and 4th to 7th (combined) presentations of the novel stimulus relative to the 1st presentation. This effect occurred whether the target appeared in conjunction with the novel or repetitive stimulus. It is not possible to definitely say whether this effect occurred because the novel stimuli slowed or enhanced responses. However, we can assume that the novel stimulus was able to attract attention, which consequently influenced RTs. Similar effects have been observed in other cognitive experimental procedures when novel elements “pop-out” and familiar items “sink-in” to the display [41]. There was also a main effect of target location. Response times in general were significantly faster when the target appeared in conjunction with the novel, relative to the repetitive, stimuli. In the paradigm of attentional cueing developed by Posner [34], faster response times at a cued location are taken to indicate that attention is orientated to the location prior to the presentation of the target, hence faster response times. The current findings show that novelty can act to unconsciously cue attention to a spatial location. This supports the theory of novel “pop-out” which argues that novel visual elements attract rapid covert shifts in attention [41, 42].

In order to obtain a single measure of the effect of the novel stimulus on responding, the difference between response times from the 1st presentation and combined 2nd-3rd presentations for targets appearing in conjunction with the novel event were calculated. This gives a simple measure of the impact of novelty on the performance of individual participants. When this statistic was compared to personality dimensions with the Tridimensional Personality Questionnaire, it was found that there was a significant negative correlation with novelty seeking. Those patients with low novelty seeking scores showed the highest impact of novelty on the responses. Novelty seeking is considered to be a trait dependent on dopaminergic tone [6] and has been found to be lower in PD patients compared to controls [13]. The negative correlation therefore seems to be paradoxical in that it may have been hypothesised that high novelty seeking individuals would show the highest impact of the novel stimuli. However, it may be that as low novelty seeking predisposes to lower behavioural responses to novelty, the initial presentation of the novel stimulus produced inhibited responding. When the level of novelty was reduced (2nd-3rd presentation) responding was returning to normal therefore giving a larger difference on the impact of novelty.

The current study found that harm avoidance scores were significantly higher in female patients with PD than male patients. We also found that harm avoidance was positively and significantly correlated with anxiety scores in our PD sample. This contributes to findings that there is a link between the temperament trait of harm avoidance and anxiety [43]. More surprising was our failure to find a link between depression and harm avoidance scores, as this link is commonly reported in nonneurological samples. One explanation maybe that depression in PD is somewhat different in its manifestation than general depression. For example it has been argued that depression in PD is indicative of a more advanced and widespread neurodegenerative illness [44, 45]. Furthermore, it has been observed that symptom profiles and responses to antidepressant medication are different in depressed patients with PD compared to general depressed patients, suggesting a different underlying pathological mechanism [30]. However, levels of depression were generally low in our PD sample, with only 15% scoring in the range of probable clinical depression, and this is an alternative explanation for our lack of association with harm avoidance scores.

## 5. Conclusions

Temperament traits in people with PD may be associated with aspects of cognitive performance and with affective disorder. In particular, we provide evidence that harm avoidance may be more linked to the presence of anxiety than to depression in people with PD. The trait of novelty seeking, which is thought to have low expression in PD, was found to be associated with performance of a cognitive task involving orientation to visual novelty. These findings extend our understanding of how temperament interacts with other manifestations of PD. 

## Figures and Tables

**Table 1:** Response times (and *SDs*) in milliseconds for the PD and control participants in the novelty attention task.

	Parkinson's		Control	
Level of novelty	Novel	Repetitive	Novel	Repetitive
1	417 (85)	425 (85)	377 (60)	383 (63)
2	409 (90)	416 (83)	370 (54)	380 (60)
3	411 (95)	415 (89)	361 (47)	371(58)
4	420 (92)	416 (88)	372 (59)	380 (56)
5	409 (94)	416 (96)	368 (60)	386 (69)
6	398 (96)	418 (89)	375 (69)	382 (71)
7	406 (99)	418 (92)	366 (55)	371 (55)

“Novel” indicates when the target appeared in conjunction with the novel stimuli and “Repetitive” indicates when it appeared in conjunction with the repetitive stimuli. The level of novelty ranges from when a novel shape was shown for the very first time (1) to when it had been shown 7 times.

**Table 2:** Correlation coefficients and *P* values for the associations between temperament dimensions in the PD sample with cognitive and affective measures.

	RT difference	Depression	Anxiety
Novelty seeking	−.505, *P* = .023	.004, *P* = .987	−.116, *P* = .625
Harm avoidance	−.238, *P* = .313	.309, *P* = .186	.508, *P* = .022
Reward dependence	.001, *P* = .996	−.378, *P* = .100	−.436, *P* = .055
